# Scales for Hearing Impairment Among Centenarians and Offspring: Implications for Cognitive Screening

**DOI:** 10.1093/geroni/igaf122.2871

**Published:** 2025-12-31

**Authors:** Angella Lee, Bradley Petrowitz, Thomas Perls, Paola Sebastiani, Stacy Andersen

**Affiliations:** Boston University, Boston, Massachusetts, United States; Boston University, Boston, Massachusetts, United States; Boston University, Boston, Massachusetts, United States; Tufts Medical Center, Boston, Massachusetts, United States; Boston University, Boston, Massachusetts, United States

## Abstract

As the need to assess cognitive function in older adults increases, it is crucial to understand how hearing may impact cognitive test performance since many tasks are administered orally. While audiometry remains the gold standard for measuring hearing loss, other methods such as self-reports are easier to collect during remote assessments and/or in large cohort studies. Centenarian (n = 291, mean age 102.2 ± 2.2 years) and offspring (n = 481, mean age 74.0 ± 7.6 years) participants from the Integrative Longevity Omics study and Longevity Consortium Centenarian Project filled out self-rated hearing ability (Likert scale from ‘excellent’ to ‘unable to hear’) and the Hearing Handicap Inventory (HHIE, range 0-40, cutoff > =10). Examiners administered a cognitive screener (i.e, Mini-Mental State Examination [MMSE]) and indicated whether hearing impairment affected the test. In stratified analyses by generation, we used linear regressions with each measure of hearing impairment adjusting for age, sex, and education to predict MMSE score. The prevalence of hearing impairment ranged from 55% to 73% among centenarians depending on the assessment modality and from 2% to 33% for offspring. Only 10.2% of centenarians and 0% of offspring were rated as hearing impaired across all three measures. Examiner-rated validity was a significant predictor of MMSE performance (p < 0.001) in both generations, while self-rated hearing was a significant predictor only among centenarians (p < 0.01). These scales capture different aspects and levels of hearing impairment and thus both research and clinical settings should consider using multiple scales to account for the impact of hearing ability on cognitive test performance.

